# Establishing and applying an adaptive strategy and approach to eliminating malaria: practice and lessons learnt from China from 2011 to 2020

**DOI:** 10.1080/22221751.2022.2026740

**Published:** 2022-01-21

**Authors:** Fang Huang, Xin-Yu Feng, Shui-Sen Zhou, Lin-Hua Tang, Zhi-Gui Xia

**Affiliations:** Chinese Center for Disease Control and Prevention (Chinese Center for Tropical Diseases Research), NHC Key Laboratory of Parasite and Vector Biology, WHO Collaborating Center for Tropical Diseases, National Center for International Research on Tropical Diseases, National Institute of Parasitic Diseases, Shanghai, People’s Republic of China

**Keywords:** Malaria, elimination, surveillance and response, strategy and approach, China

## Abstract

On 30 June 2021, China was certified malaria-free by the World Health Organization. In this study, the evolution, performance, outcomes, and impact of China’s adaptive strategy and approach for malaria elimination from 2011 to 2020 were analysed using 10-year data. The strategy and approach focused on timely detection and rapid responses to individual cases and foci. Indigenous cases declined from 1,308 in 2011 to 36 in 2015, and the last one was reported from Yunnan Province in April 2016, although thousands of imported cases still occur annually. The “1–3–7” approach was implemented successfully between 2013 and 2020, with 100% of cases reported within 24 h, 94.5% of cases investigated within three days of case reporting, and 93.4% of foci responses performed within seven days. Additionally, 81.6% of patients attended the first healthcare visit within 1–3 days of onset and 58.4% were diagnosed as malaria within three days of onset, in 2017–2020. The adaptive strategy and approach, along with their universal implementation, are most critical in malaria elimination. In addition to strengthening surveillance on drug resistance and vectors and border malaria collaboration, a further adapted three-step strategy and the corresponding “3–3–7” model are recommended to address the risks of re-transmission and death by imported cases after elimination. China’s successful practice and lessons learnt through long-term efforts provide a reference for countries moving towards elimination.

## Introduction

Although an estimated 229 million malaria cases and 409,000 deaths in 87 malaria-endemic countries have been recorded recently [1], global progress in reducing the overall morbidity and mortality from malaria has been achieved in the past two decades. Many countries have made great strides in controlling malaria or moving towards elimination, and 40 countries and territories have been granted malaria-free certification from the World Health Organization (WHO), including China, in 2021 [2].

Malaria was historically prevalent in China, and was recorded in oracle bone inscriptions as early as 3,000 years ago [3]. During the 1940s, at least 30 million malaria cases, 1% of which resulted in death, occurred annually and more than 70% of counties had malaria transmission [3]. Malaria has been managed as a notifiable disease in China since 1956 [4,5]; meanwhile, in the 1950s, China established professional organizations and carried out baseline investigations and malaria prevention and control practices in targeted regions [6]. In the following 20 years, malaria pandemics in the 1960s and 1970s were controlled through a comprehensive strategy with a focus on mass drug administration (MDA), mass protection, and intranational cooperation [7], and the incidence declined from 1,500/100,000 in early 1960s to 257/100,000 in 1979. Since 1980, malaria had been characterized by a sustained decline in incidence as a result of various comprehensive measures based on vector habits in receptive areas with different *Anopheles* spp. [8–10]. By 1999, malaria cases were less than 30 thousand and incidence reduced to appropriately 3/100,000, a total of 1,321 counties or cities had been confirmed to have achieved “basic malaria elimination” [6,11], with incidence rates less than 1/10,000 for three consecutive years, and falciparum malaria had been eliminated except in Yunnan and Hainan Provinces. From 2000 to 2009, the strategy was focused on early diagnosis and appropriate treatment, and integrated measures including blood testing, vector control, and health education for targeted risk populations were strengthened and supported by the Global Fund [12–14]. The epidemic resurgence and local outbreaks of malaria in central China emerged between 2001 and 2006, and malaria cases have rapidly increased tens of thousands of times, with more than 40 thousand cases reported in 2006. This resurgence was mainly caused by climate warming leading to extending malaria transmission with increasing vectorial capacity of *An. sinensis* and low capacity of diagnosis leading to accumulation of infectious sources, and was controlled by target MDA and case management [15,16]. Local transmission of *Plasmodium falciparum* had occurred only in Yunnan Province since 2009. An incidence rate of more than 1/1,000 had been reported in four counties in the entire country [17], but 95% of the counties in all 24 malaria-endemic provinces had reported incidence rates of less than 1/10,000, with only 87 counties reporting more than 1/10,000 [18]. Most malaria-endemic areas had maintained incidence at less than 1/10,000 for several decades.

Comparing with the WHO’s classification criteria for the phases from control to elimination [19], which adopted less than 1 case/1,000 risk population per year as an indicator of the transition from pre-elimination to elimination, China used 1/10,000 as an indicator for malaria stratification in the elimination phase. In 2010, in response to the global malaria eradication initiative proposed at the United Nations (UN) Millennium Development Goals High-Level Meeting, the Chinese government formulated and launched the “National Action Plan for Malaria Elimination,” with the goal of eliminating malaria nationwide by 2020 [20]. China adhered to the principles of “prevention first, scientific control, adaptative measures, and classified guidance” combined with the mechanisms of “government leadership, multisector cooperation, and whole-society participation” to promote the progress of the national elimination programme. The number of indigenous malaria cases and the associated disease burden reduced dramatically in the following several years. The last indigenous case was reported from Yunnan Province in April 2016 [21]. A subnational verification for malaria-free status in individual provinces led by the government was initiated in 2016 [22]. By 2020, all 24 provinces that were historically malaria-endemic had received a national designation of malaria elimination. No indigenous cases have been reported since 2017 [21]. In November 2020, the Chinese government submitted an official request for malaria elimination certification from the WHO. On 30 June 2021, the WHO certified China as malaria-free, a major milestone for a country with a population of 1.4 billion people [23].

The strategy and approach to eliminating malaria in different phases or stages in China were continuously adjusted and improved to adapt to the national elimination goal. Several studies demonstrated performance and experiences in the beginning stage of malaria elimination in China [24–27]; however, the evolution of the elimination process and its whole picture reviewed on an evidential basis during the journey to malaria elimination in China have not yet been clearly and systematically documented through a 10-year retrospective analysis using the national data.

Through objectively analysing and evaluating the core strategy and approach and their implementation, this study aims to accurately condense the successful and replicable experience of malaria elimination in China, and the lessons learnt. Challenges and potential solutions in preventing the re-establishment of malaria in the post-elimination phase are identified and presented.

## Materials and methods

*Data collection*. Legislation and regulations relevant to malaria elimination in China from 2010 to 2020 were collected and reviewed. The updates and revisions of strategic plans and technical guidelines were analysed to describe the evolution of the adaptive strategy and approach undertaken to achieve elimination. Malaria data, including data on *Plasmodium* species, case classification, source of malaria, date of illness onset and diagnosis, the implementation of interventions, and outcome and impact indicators, were collected via the National Notifiable Infectious Diseases Surveillance System and the Information System for Parasitic Disease Control and Prevention. Data from Hong Kong, Macao, and Taiwan were not included.

Malaria cases were confirmed according to malaria diagnostic criteria in China [28]. According to the national surveillance guideline of 2015 [29], all reported malaria cases were classified into five categories: indigenous, imported, relapsing/recrudescent, introduced, and induced. Indigenous malaria is defined as malaria transmitted by an *Anopheles* mosquito in Chinese territory, without evidence of importation or introduction. Here, an introduced case is defined as a case contracted locally, with strong epidemiologic evidence linking it directly to a known imported case (first-generation local transmission). The exclusion criteria for indigenous malaria based on the epidemiological history were as follows: cases reported in nonreceptive areas; cases reported during the nonmalaria transmission season and during the incubation period before onset; *P. falciparum* malaria cases in areas lacking a transmission vector for *P. falciparum*; no indigenous or introduced cases caused by the reported species for at least three years; or clear evidence of another classification of the case.

*In vivo* therapeutic efficacy of the antimalarial drug against malaria infections were evaluated from the first day of treatment to the specified last day of the follow-up period following the WHO guidelines for therapeutic efficacy monitoring [30].

*Data analysis*. Data were analysed using Microsoft Excel (version 2010). Statistical analysis was performed using SAS software (version 9.4). The chi-squared test was used to evaluate differences among the sub-groups and Fisher’s exact test was used if 25% of the cells had expected counts less than 5. Box plots were visualized using GraphPad Prism 8.4.3 (GraphPad Software, LLC., San Diego, CA, USA). A *P* value less than 0.05 was considered to be statistically significant.

## Results

## Development of strategy and approach, technical innovations, and supporting systems

As malaria shifted from high to low transmission in China, the strategy and approach to targeting malaria elimination nationwide were developed. According to the evolving malaria situation, China continuously modified and optimized its strategic and technical elements and supporting systems to identify and subsequently fill the programmatic gaps [29].

*Case- and focus-centred comprehensive strategy.* Several innovative elements of the strategy for malaria elimination were proposed in the Action Plan. First, the incidence rate of 1/10,000 was used as the indicator and criteria for stratification, and malaria-endemic areas were categorized at the county level, with different targeted interventions and objectives [18]. Second, the goal would be achieved in a staged manner, with the first stage to be completed by 2015 in most malaria-endemic areas with the relatively low transmission, then the second stage to be achieved by 2020 in the highest transmission areas in Yunnan Province [20]. Third, strategy was tailored for elimination from population-based to case- and focus-centred comprehensive interventions. The term “focus,” which is used to describe natural villages with a defined and circumscribed area and population in which malaria cases are detected, was first formulated in the Action Plan [20].

**Figure 1.**
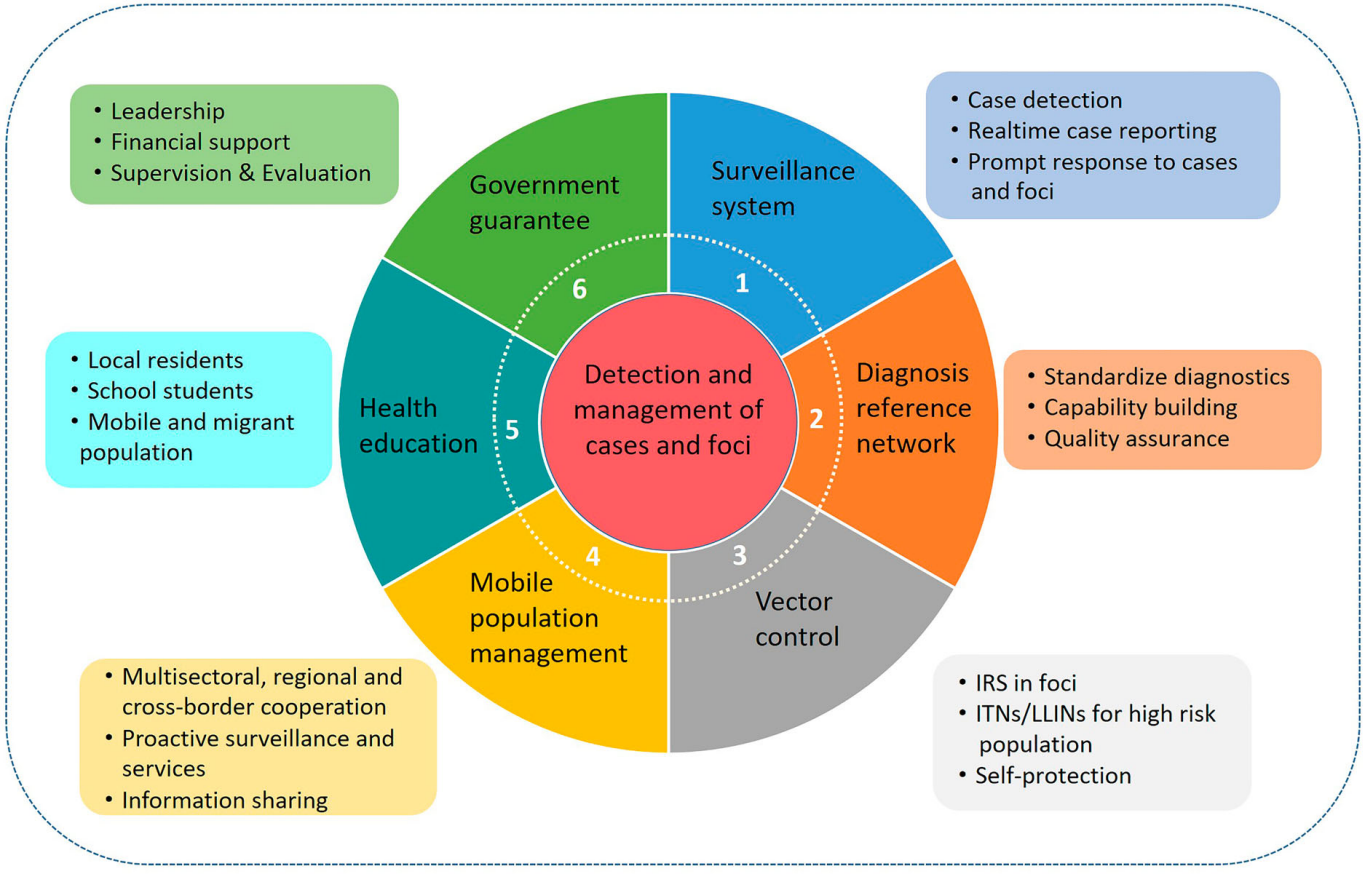
Core components of strategy stated by the National Action Plan for Malaria Elimination in China (2010–2020).

The core strategy in the Action Plan was the detection and management of cases and foci, combining a surveillance and response system with a real-time reporting system and case diagnosis reference networks, vector control (including indoor residual spraying [IRS] in foci, long-lasting insecticidal net [LLIN] distribution in high risk areas, and personal protection), malaria management among migrant populations (including information sharing between sectors, and joint and proactive service delivery), health education (target populations such as the general public, populations at high risk, students), and strengthening government guarantees (including leadership, responsibilities, financial support, affordable drug policies, capability building, supervision, and evaluation) (Figure 1).

*Initiation of the 1–3–7 approach*. The 1–3–7 approach was initially conceptualized in the 2011 Technical Scheme [31] and required all suspected, clinically diagnosed, and confirmed malaria cases to be reported through the Disease Reporting Information System of the Chinese Center for Disease Control and Prevention (CDC) within 24 h (“1 day”) and appropriate treatment to be administered following the national antimalarial guidelines. An epidemiological case investigation should then be completed within “3 days” of reporting and focus investigations and responses should be completed within 1 week (“7 days”), to evaluate the potential risk of transmission and enable response actions to be taken accordingly. The 1–3–7 approach as a formal term was published in Chinese in *Malaria Control and Elimination in China* in 2013 [32] and in English in the journal of PLoS Medicine in 2014 [33]. Since then, this approach has been widely rolled out nationwide [24,34–36].

*Adaption of suspected patients and case diagnosis*. In the early stage of elimination from 2010 to 2014, “suspected malaria patients” mainly referred to cases with one of “three types of fever”: fever with typical malaria symptoms, fever with atypical malaria symptoms, or fever of unknown cause [30]. After 2015, “suspected patients” referred to cases with one of “four types of fever”: febrile patient with a travel history in a country where malaria is endemic, or having a blood transfusion in the past two weeks, or with a history of malaria, or with an unknown fever cause [29]. In China, all suspected malaria patients are required to undergo laboratory testing in the local CDC or the nearest medical centre.

According to the WHO criteria for case detection and confirmation, microscopy or rapid diagnostic test (RDT) is required in the malaria elimination phase [37]. In China, both clinically diagnosed and confirmed malaria cases should be reported by health providers [32,33]. Meanwhile, cases must be first confirmed with species by the county CDC where the case was reported. Case investigation, including confirmation, is completed within three days by the county CDC. Subsequently, the result is rechecked and reconfirmed by the provincial diagnosis reference laboratory.

**Figure 2.**
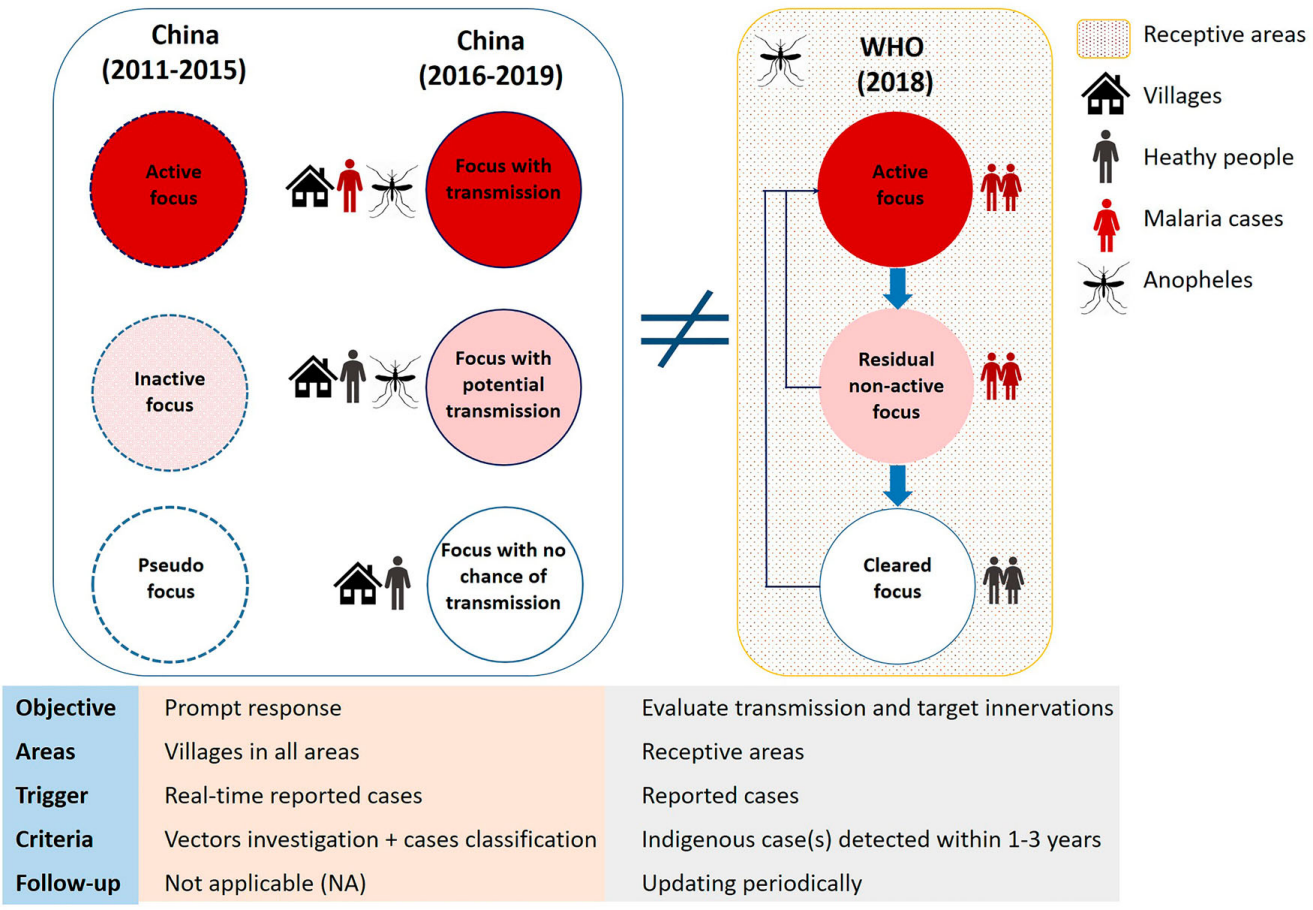
Classification of malaria foci in China compared with the WHO guidelines.

*Adaption of malaria focus definition and classification*. In China, a focus was defined as a natural village in which a malaria case occurred. Natural village is the basic community with relative independence in space and administrative management in China. There were two versions of “focus classification” in China (Figure 2). The first version was formulated in 2010 along with the Action Plan and the Technical Scheme [31]. Foci were classified into three types: active focus, inactive focus, and pseudo focus. These were updated to transmission focus, potential transmission focus, and focus with no chance of transmission in the second version in 2016 [29]. These classifications of foci were applied across the entire country, including in receptive and nonreceptive areas, with a real-time reported case as the key criteria. The case classification was another component of focus determination. A prompt response with various interventions was the main goal of the focus investigation. However, periodic updating was not adaptive in terms of focus classification and management in China [29].

**Figure 3.**
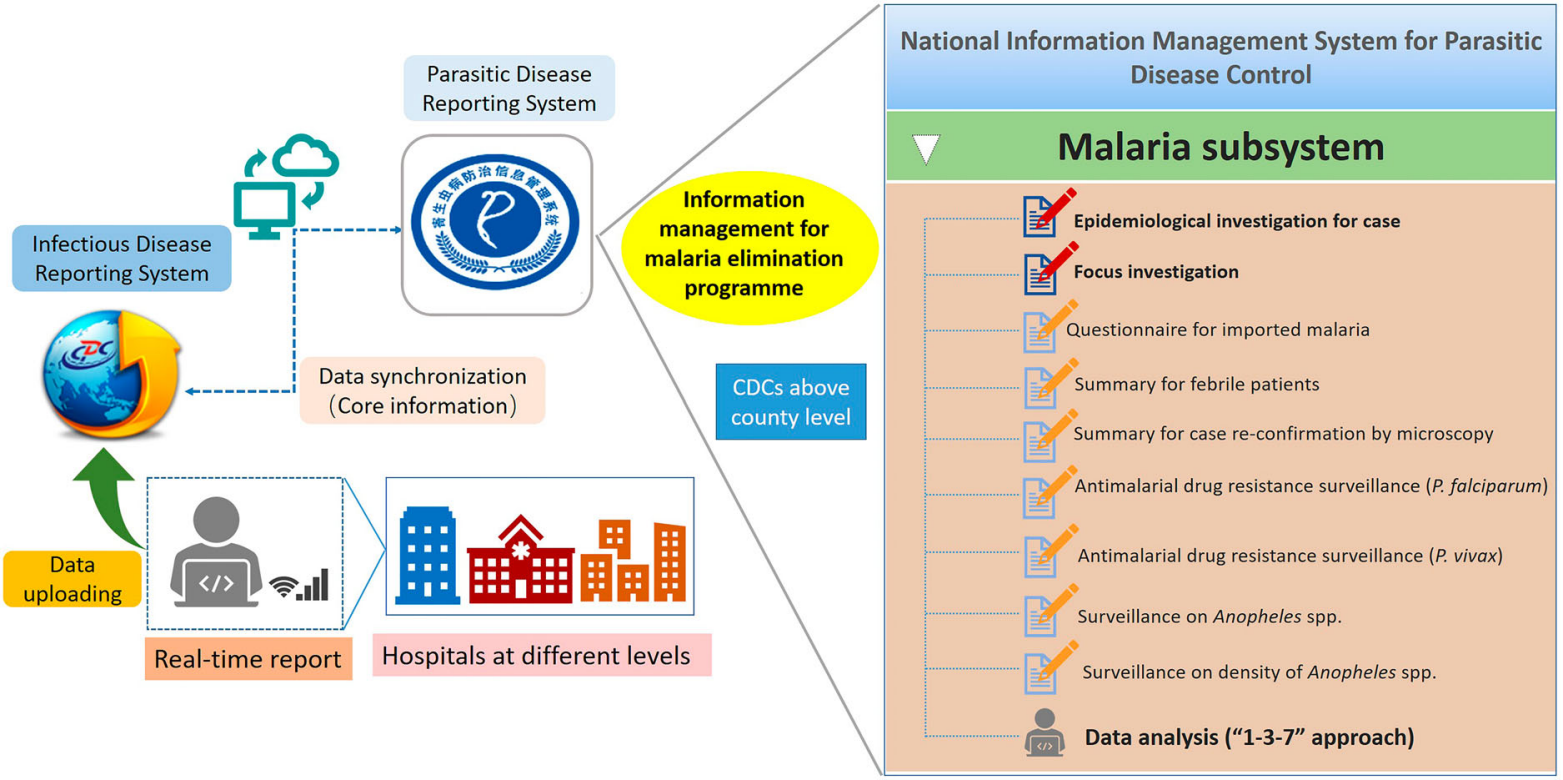
Web-based information systems for the Malaria Elimination programme in China.

*Construction and reinforcement of supporting system*. Hard copies of malaria data have been aggregated in a central repository until the early portion of this century. The “malaria subsystem” was added to the web-based National Information Management System for Parasitic Disease Control in 2011, which included basic information, information about the epidemiological investigations, focus investigation and response actions, and other related malaria surveillance data (Figure 3). Owing to real-time case reporting, timely review and feedback, and routine data analysis and information exchange, this system has promoted rapid and accurate interventions and responses.

The national malaria diagnosis reference laboratory network was initiated in 2011 and is responsible for professional training, capability evaluation, quality assurance, and internal and external quality assessments to ensure diagnostic quality. Currently, this network covers 24 historically malaria-endemic provinces and one non-endemic province (Supplemental Data) [38]. Supported by this network, blood tests are required with blood collected before the initiation of treatment and are reviewed by staff at multiple levels. All positive and at least 10% of negative slides must be verified at county, prefectural, and provincial laboratories.

### Outcomes and impacts of strategy and approach

**Figure 4.**
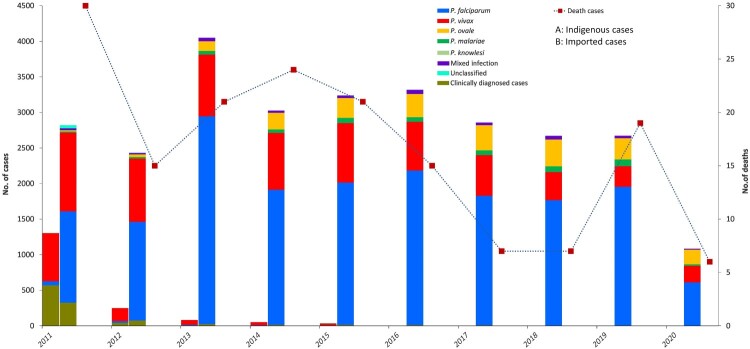
Reported malaria cases (indigenous and imported) and deaths in China, 2011–2020.

*Reported cases of malaria and population at high risk.* A total of 30,278 malaria cases were reported from 2011 to 2020, with 165 deaths. Of these, 1,732 were indigenous cases, 28,173 were imported cases, nine were induced cases, five were long-incubation *P. malariae* cases, and four were introduced *P. vivax* cases. Imported cases remained in the thousands annually, while indigenous cases declined from 1,308 in 2011 to 36 in 2015 (Figure 4). The last indigenous case, one *P. vivax* patient, was reported from Yunnan Province in April 2016 [21]. Since 2017, no indigenous malaria cases have been reported, but thousands of imported cases have occurred annually. Except 1,223 clinically diagnosed cases, 29,055 cases among all the reported cases were confirmed by blood examination, including 17,960 *P. falciparum* cases, 7,818 *P. vivax* cases, 2,257 *P. ovale* cases, 558 *Pmalariae* cases, 374 mixed infection cases, 2 *P. knowlesi* cases, and 86 unclassified cases. The distribution of *Plasmodium* spp. between imported cases and indigenous cases was different (*P *< 0.001). *P. vivax* was predominant among the indigenous cases, whereas *P. falciparum* accounted for 51.4%–73.1% of imported cases during 2011–2020 (Figure 4). In addition, dozens of deaths related to malaria have been reported annually.

With the decrease in the number of malaria cases in China, the number of people at risk of malaria infection has markedly decreased, from 281 million in 2011 to 0.3 million in 2020. The numbers of blood examinations by microscopy and RDTs in routine surveillance have declined accordingly, although the positivity rate for blood examinations has greatly fluctuated, which might be caused by the varying attitude of patients to seeking medical care and the different capabilities for malaria diagnosis (Table 1). A total of 5.95 million people in malaria foci have been protected by IRS during this time (Supplemental Data). Insecticide-treated bed nets (ITNs) and LLINs have been used as an important supplementary vector control strategy, promoting malaria elimination since the Action Plan was launched. However, vector control interventions were not the same in different receptive and transmission areas in China. ITNs and LLINs were mainly implemented in the areas with a high risk of malaria transmission, whereas IRS was the key intervention for foci response to interrupt transmission or prevent potential transmission. China distributed 2.25 million ITNs and LLINs in endemic provinces, with the majority distributed in Yunnan and Hainan provinces, between 2011 and 2020.

**Table 1.** Risk population and blood tests in China, 2011–2020.

Year	Total population (10,000)	Risk population (million) ^#^	No. of blood examinations for routine malaria surveillance (10,000) *	No. of positive blood examinations	Annual blood test rate (%) ^※^	Positive rate (%)
2011	134,735	281	740.4	3,491	0.550	0.05
2012	135,404	171	689.1	2,556	0.509	0.04
2013	136,072	8.18	562.2	4,002	0.413	0.07
2014	136,782	8.57	441.5	2,978	0.323	0.07
2015	137,462	5.51	407.7	3,160	0.297	0.08
2016	138,271	0.35	320.6	3,236	0.232	0.10
2017	139,008	0.42	233.2	2,796	0.168	0.12
2018	139,538	2.97	191.6	2,597	0.137	0.14
2019	140,005	0.60	168.6	2,612	0.120	0.15
2020	138,111	0.30	127.4	1,084	0.092	0.08

^#^ It is the population in active foci and foci with risk of transmission.

*The number of samples detected by microscopic examination and RDTs is included.

^※^ The number of people at risk varies greatly according to the number of people in active foci and foci with risk of transmission, and the annual blood examination rate calculated with the risk population as the denominator is easily affected.

*Outcomes of the case- and focus-centred strategy and approach*. Between 2013 and 2020, of all the malaria cases detected and notified, 100% were reported within 24** **h, and a total of 21,839 (94.5%) epidemiological case investigations were completed within three days of case diagnosis. A total of 21,579 (93.4%) foci investigations and responses were performed within seven days. Since the implementation of the Action Plan, malaria case detection has included passive case detection (PCD), reactive case detection (RACD), and proactive case detection (PACD). PCD primarily involves blood tests performed in public/private hospitals and military departments and a total of 37.5 million patients were tested during 2011–2020 with 28,452 positive detections. The majority of 32 RACD-positive patients detected from 105,384 residents of foci and co-travellers and 35 PACD-positive cases from 1,215,309 at risk populations were asymptomatic cases. The positive rates of RACD and PACD were lower than the PCD positivity rate (*P *< 0.001), as patients detected by PCD, had symptoms of suspected malaria or/and a travel history to malaria-endemic regions. In addition, all the positive cases detected by RACD and PACD were required to be confirmed in the malaria diagnosis reference laboratory.

**Figure 5.**
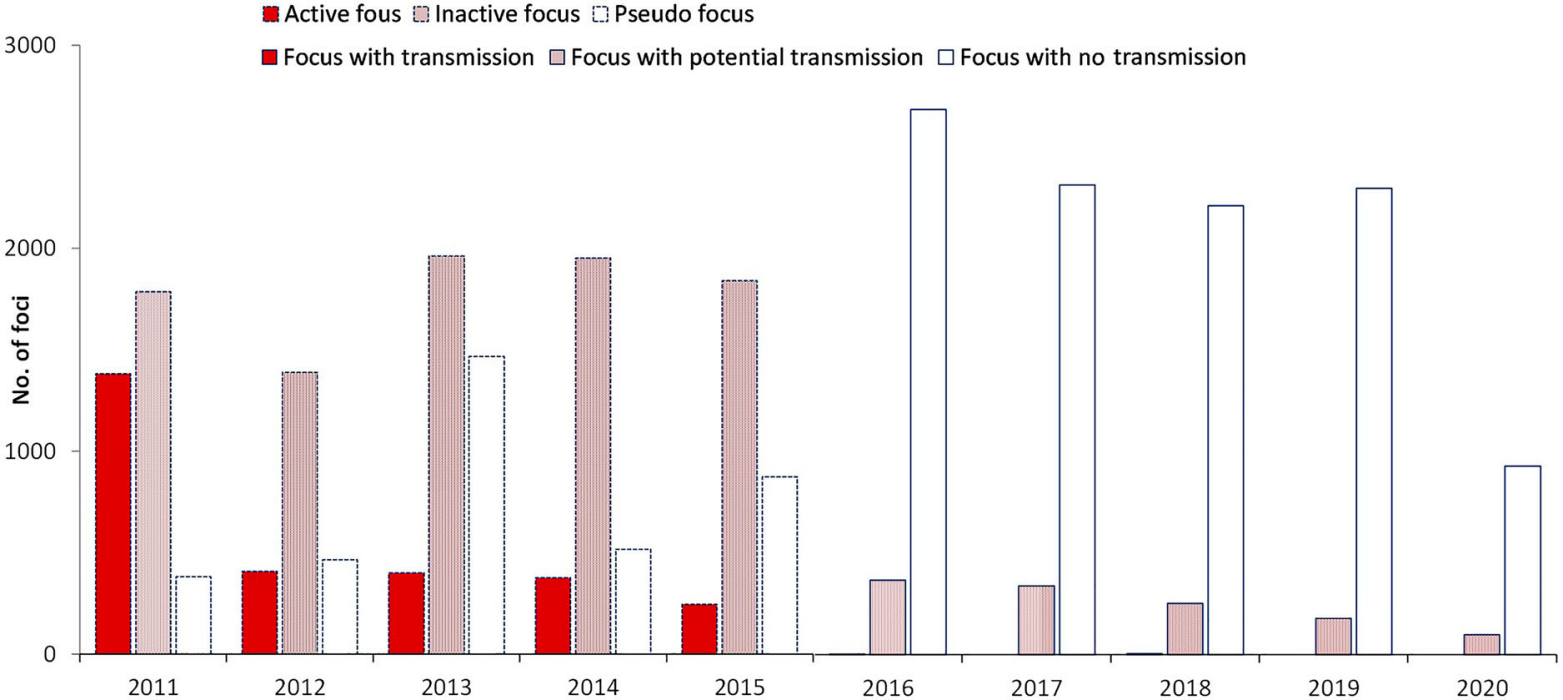
Number of malaria foci classified in two stages, 2011–2020.

A total of 27,558 foci were identified during 2011–2020. Focus classification was recorded considering two periods; a total of 15,436 foci, including 2,810 active foci, 8,926 inactive foci, and 3,700 pseudo foci, were reported in the first period (2011–2015), and 12,122 foci, including five foci with transmission, 1,232 foci with potential transmission, and 10,435 foci with no chance of transmission, were reported in the second period (2016–2020) (Figure 5).

**Figure 6.**
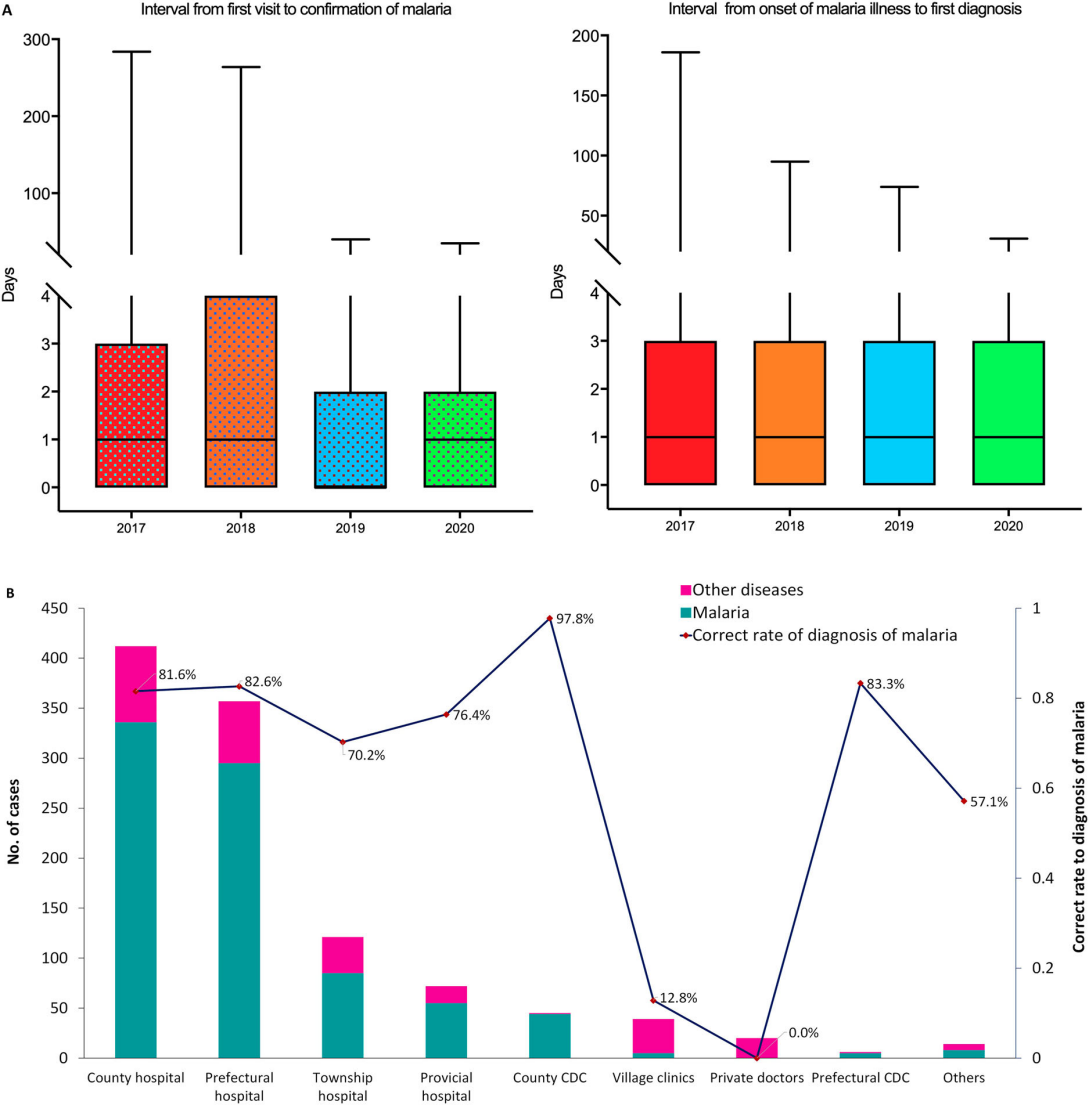
Patients’ awareness of health care and malaria diagnostic capacity in different facilities in China. (A) Interval from malaria illness onset to first visit to a healthcare facility and interval from first visit to diagnosis of malaria, 2017–2020. (B) Distribution of healthcare facilities receiving first visit malaria patients and their correct diagnosis rates, 2017–2020.

*Disease awareness, access to health care, and diagnostic capacity*. From 2017 to 2020, a total of 9,287 malaria cases were reported within 24 h; 81.6% (7,582/9,287) of patients accessed health care, with the first visit within 1–3 days of illness onset. Of these, 3,126 consulted a doctor on the day of onset of illness (Figure 6(A)). The median interval from illness onset to the first visit was 1 day. However, a number of patients sought medical care several months after the onset of illness. A total of 4,384 malaria cases were diagnosed by microscope or RDTs at the first visit, and 7,375 cases were diagnosed within three days. The median interval from the first visit to diagnosis was also 1 day (Figure 6(A)). With respect to the time from the onset of symptoms to diagnosis, more than half of patients (58.4%, 5,426/9,287) were diagnosed with malaria within three days of onset. In 2020, a total of 1,086 cases were reported; 37.9% of the cases were diagnosed in county hospitals, followed by prefectural hospitals (32.9%) and township hospitals (11.1%) (Figure 6(B)). Only approximately 10% of the cases were diagnosed by CDCs at the provincial and county levels. Moreover, not all cases were diagnosed correctly as malaria on the first visit, with 23.3% (253/1,086) of cases diagnosed as other diseases. The reason for misdiagnosis was lower awareness of malaria of first visit doctors, with them not requesting malaria detection using microscope or RDTs in the management of suspected patients. Malaria detection capability differed significantly between healthcare facilities (*P *< 0.001). County CDCs had the highest rate of correct diagnosis, at 97.8%. A rate of more than 80% was achieved by prefectural hospitals and CDCs, whereas malaria diagnosis accuracy was relatively low in village clinics and other facilities (Figure 6(B)).

*Drug resistance surveillance and entomological investigations*. During 2016–2020, malaria was undergoing a transition from pre-elimination to elimination, and the target patients for therapeutic efficacy study were changing from indigenous cases to imported cases, while the latter were more mobile and difficult to follow-up, therefore, the therapeutic efficacy of dihydro-artemisinin-piperaquine (DHA-PPQ) and chloroquine (CQ) for the treatment of uncomplicated *P. falciparum* and *P. vivax* infection, respectively, were only evaluated in enrolling 39 *P. falciparum* and 21 *P. vivax* patients, with cure rates >90% and 100%, respectively. Meanwhile, molecular markers were used to amplify the artemisinin resistance genes *Pfkelch* 13 on 2,483 imported malaria samples by passive or active detection. A total of 54 non-synonymous mutations in the *Pfkelch* 13 propeller domain were confirmed, with a prevalence of 3.5%, and the most common mutation was A578S, with a proportion of 16.1%, followed by Q613E (6.9%) [39]. There were no cases reporting artemisinin treatment failure, although a few patients carried *Pfkelch* 13 mutations associated with artemisinin resistance [40,41].

Entomological investigations have been implemented at the sentinel sites across the country, showing that *Anopheles sinensis* was the most widely distributed species, followed by *An. anthropophagus* (synonym: *Anopheles lesteri*), *An. minimus* s.l., and *An. dirus* s.l*.* (Supplemental Data). The distribution of *Anopheles* spp. differed across the provinces of China. *An. anthropophagus* was identified in Guizhou, Sichuan, Hainan, and Liaoning provinces; *An. minimus* s.l. was detected in Yunnan and Hainan provinces; *An. dirus* s.l. was only found in Hainan Province and *An. maculatus* was identified in Tibet.

## Discussion

In the past 10 years, 17 countries have achieved zero local malaria transmission, eight of which have been certified malaria-free by the WHO [42]. China was awarded malaria-free status from the WHO following a 70-year effort, which was a major milestone for the world’s most populous nation. The elimination of malaria is a comprehensive public and social project and cannot be achieved independently by health departments alone. The commitment and leadership of the government have provided sustained financial and human resources to ensure the implementation of the national malaria programme in China (Figure 1) and the Ministry of Health has established high-level inter-ministerial joint meetings to accelerate the Action Plan through multisectoral, multiregional, and multidisciplinary cooperation, which ensures that all activities and measures are performed in time. In addition, China has made remarkable economic progress following reforms and opening-up since the 1980s [43,44], and improvements in living conditions and medical insurance coverage in rural and remote areas have greatly promoted progress towards malaria elimination. Traditional Chinese medicine combined with modern medicine (Western medicine) has made a remarkable contribution; the discovery, extraction, purification, and chemical characterization of artemisinin from *Artemisia annua* by Tu Youyou and her team led to a Nobel Prize in Physiology and Medicine in 2015 [45].

China launched its Action Plan utilizing existing tools in 2010, with the goal of elimination by 2020 [20]. The Action Plan, together with accompanying guidelines, reflected the WHO’s global guidelines for elimination while incorporating strategic and technical adaptations that allowed for more timely, cost-efficient, targeted, and tailored approaches reflecting local transmission dynamics [46]. A few points were differently defined compared with the WHO guideline. For example, the WHO 2012 country classification includes four phases on the path to malaria elimination [19]. This classification is indicative of the transition between phases, with slide positivity rates (SPRs) or RDT positive rates of less than 5% among fever patients as an indicator of the transition from control to pre-elimination and less than 1 case/1,000 risk population per year as an indicator of the transition from pre-elimination to elimination. However, the classification criteria for the phases in China differed [20]. When China launched the Action Plan in 2010, the major task of malaria reduction had been achieved, and most of the counties were already in the elimination phase according to WHO criteria. In contrast, China established a much more robust requirement based on the long-standing fight against malaria, which laid a solid foundation for eliminating malaria in the country. This indicated that the effective interventions adopted in the control phase by drastically reducing the incidence rate will ultimately make a great contribution to the elimination of malaria in the country. Secondly, according to the WHO definition, a “focus” is defined as a circumscribed area situated in a currently or formerly malarious area that contains the epidemiological and ecological factors necessary for malaria transmission [36]. In China’s strategy, it was defined as a natural village in which a malaria case occurred, differing from the WHO definition. The key criterion and role for foci in China were a quick response for all reported cases and the prevention of potential risk of re-establishment.

The case- and focus-centred comprehensive strategy and 1–3–7 approach adopted in China proved crucial in malaria elimination; nevertheless, this was not performed perfectly in the earlier elimination stage. First, clinically diagnosed and unclassified cases occurred in some areas, particularly in non-endemic provinces and especially in 2011 [47], because blood samples were not collected before infected patients took antimalarial drugs. After further training and refresher training on malaria diagnosis through the malaria reference diagnosis network, the capability for malaria detection improved dramatically. Secondly, although asymptomatic cases were identified through RACD and PACD, the prevalence of asymptomatic cases with different diagnosis methods was not requested by the malaria-specific reporting system. The role of asymptomatic cases in malaria elimination was not clearly understood, although the goal has now been achieved. Thirdly, a small number of patients were still lost to follow-up with the timeframe of the 1–3–7 approach, especially those from mobile or migrant populations. More importantly, almost half of malaria patients were not detected within three days of illness onset in spite of an excellent 1–3–7 performance. In response to the relatively poor awareness of timely access to medical treatment and misdiagnosis at the patients’ first visit, the National Health Commission organized routine case analyses and notifications, and initial and refresher training on malaria have been conducted at all levels to improve doctors’ awareness and capability in detecting malaria and to provide health education to influence the behaviour of patients in seeking medical treatment.

China has been certified as malaria-free; nevertheless, it remains a challenge to prevent re-establishment in the post-elimination phase. First, the case- and focus-based malaria surveillance and response system, following its effective strategy and approach, must be maintained, with strict timelines and quality indicators for timely detection and prompt response [48]. Secondly, a national antimalarial surveillance network has been established since 2016 [39], which is gradually playing an increasingly important role in the post-elimination phase. This is especially with regard to genotyping drug resistance genes and following subjects travelling from areas with high malaria endemicity, through integrated drug efficacy surveillance (iDES) to ensure timely and complete cures [39]. Thirdly, vector surveillance, in particular of insecticide resistance and selection for use, should be continuously strengthened [49]. ITNs/LLINs and IRS, as major vector control measures, have played important roles in the control and elimination of malaria in China [50,51]. In the post-elimination stage, IRS remains the major tool used in foci where transmission has occurred or where the potential for transmission exists, to interrupt transmission or prevent potential transmission by quickly eliminating *Anopheles* mosquitoes. The last and the most crucial effort is the management of cross-border malaria, which remains a threat, especially Yunnan Province along the China–Myanmar border [52–54]. Malaria elimination has been achieved on the Chinese side; however, human movement across the border and a lack of barriers for malaria vectors heighten the risk of introduction and re-establishment of malaria [55]. Multisectoral collaboration and international cooperation between border countries with endemic malaria should be sustained [52].

Given that the risks in the post-elimination phase are malaria re-establishment, and severe cases and death caused by imported malaria, the three-step strategy “traveller- and border- centred proactive and multisectoral health care, case-based timely detection and prompt response, and scientific and effective delivery of appropriate interventions and treatment” should be adopted as core. Multisectoral cooperation among health, customs, business, and tourism, etc., should be consolidated to maintain the capacity to provide proactive malaria services for outbound travellers and timely detection of inbound malaria infections. If cases are detected, rapid, scientific interventions should be taken to prevent secondary transmission and severe illness and death. Another key point that should be considered is that the 1–3–7 approach does not provide a duration requirement for case detection before diagnosis. If a patient cannot be identified through the 1–3–7 approach, re-establishment is high risk and the prognosis of the patient is negatively affected. According to the biological life cycle of parasite, the mature gametocytes, particularly of *P. vivax*, can develop as early as three days after illness onset and then grow to mature sporozoites in mosquitoes in around 10 days, ready to begin a new transmission [56,57]. Therefore, the necessary modification of the 1–3–7 approach is recommended accordingly in the post-elimination phase, with an emphasis on case diagnosis within three days after illness onset, case reconfirmation and epidemiological investigation completion within three days after diagnosis, and foci investigation and response completion within seven days after diagnosis. This 3-3-7 approach is a scientific method for improving the timeliness of patients seeking medical care and the capacity of local health facilities to diagnose malaria, in the prevention of the re-establishing of malaria.

According to WHO’s World malaria report 2021 [58], the COVID-19 pandemic has disrupted malaria services, leading to a marked increase in cases and deaths. Despite China has achieved national malaria elimination, the pressure of imported malaria persists even amid regular prevention and control of COVID-19. In particular, the lack of awareness of malaria diagnosis and referral by medical staff in quarantine places might cause the missed or delayed diagnosis and treatment of malaria among inbound travellers. Meanwhile, due to the incubation of malaria infection, it might be ignored when malaria patients do not become ill during the quarantine, which will increase the risk of severe malaria and re-transmission. Therefore, malaria health education among entry personnel, the awareness of malaria diagnosis of medical staff, and malaria screening with RDTs should be strengthened in the quarantine places. In addition, the procedures for malaria detection, reporting, and blood sample submission and review should be clarified. At the same time, it is necessary to further strengthen the tracking of inbound passengers with the longest incubation period of malaria after the quarantine is lifted, to ensure the timely detection and appropriate response of each imported case and potential focus of 3-3-7 approach.

## Conclusion

China’s experience with malaria elimination has fully demonstrated that malaria can be eliminated, despite a historical rate of 30 million cases per annum. An adaptive and practical strategy and approach along with a strong surveillance and response system have been key in malaria elimination. The WHO has updated its Global Technical Strategy for Malaria 2016–2030 [59], which outlines the critical requirements for achieving and maintaining elimination, such as a national case-based surveillance system, quality data management, and robust human and financial resources to generate, analyse, and utilize high-quality data for making decisions and tailoring responses. Malaria-endemic countries moving towards elimination may establish their own strategy, approach, and supporting systems based on existing tools, with China as a reference model in terms of its successful experience and lessons learnt.

## Supplementary Material

Supplemental Material
