# Supplementary material for: Establishing and applying an adaptive strategy and approach to eliminating malaria: practice and lessons learnt from China from 2011 to 2020
Source: Emerg Microbes Infect. 2022 Jan 21;11(1):314–25. doi: 10.1080/22221751.2022.2026740 (PMC8786258; doi:10.1080/22221751.2022.2026740)
Supplement: Supplemental Material [file TEMI_A_2026740_SM0818.zip › Suppl files/Supplemental file 2.docx]

**Supplemental Table S1 Number of ITNs and LLINs distributed and populations protected by indoor residual spraying in China, 2011–2020**

|  | **2011** | **2012** | **2013** | **2014** | **2015** | **2016** | **2017** | **2018** | **2019** | **2020** | **Total** |
| --- | --- | --- | --- | --- | --- | --- | --- | --- | --- | --- | --- |
| No. of ITNs/LLINs distributed (10,000) | 184.08 | 25.16 | 5.89 | 1.99 | 2.96 | 2.66 | 1.13 | 0.60 | 0.18 | 0.21 | 224.85 |
| No. of populations protected by IRS (10,000) | 104.40 | 109.22 | 44.76 | 50.49 | 169.72 | 27.21 | 35.27 | 16.12 | 20.66 | 17.46 | 595.32 |
