# Supplementary figures and images for: Establishing and applying an adaptive strategy and approach to eliminating malaria: practice and lessons learnt from China from 2011 to 2020
Source: Emerg Microbes Infect. 2022 Jan 21;11(1):314–25. doi: 10.1080/22221751.2022.2026740 (PMC8786258; doi:10.1080/22221751.2022.2026740)

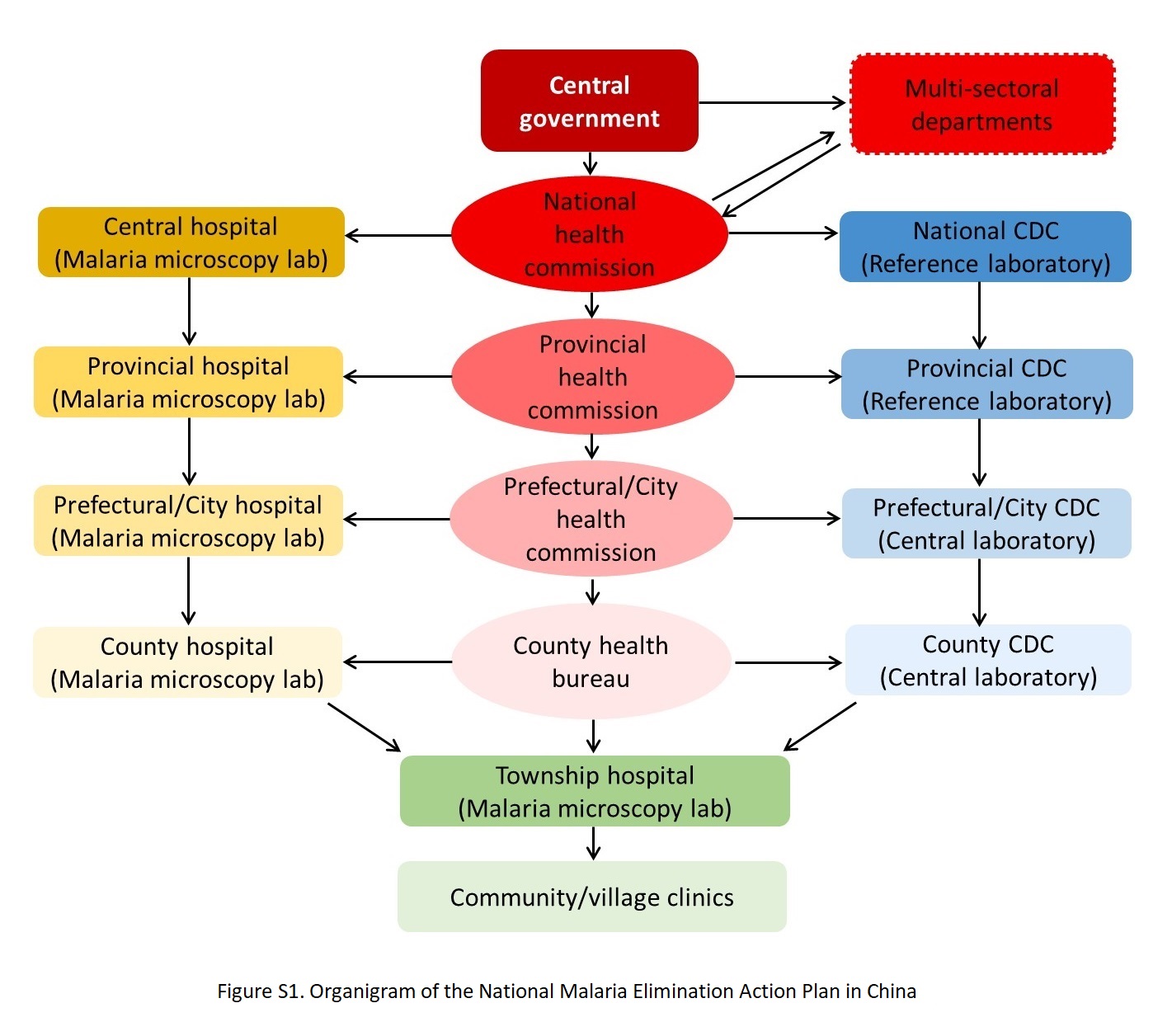

Supplement: Supplemental Material [file TEMI_A_2026740_SM0818.zip › Suppl files/Supplemental file 1-rev.jpg]

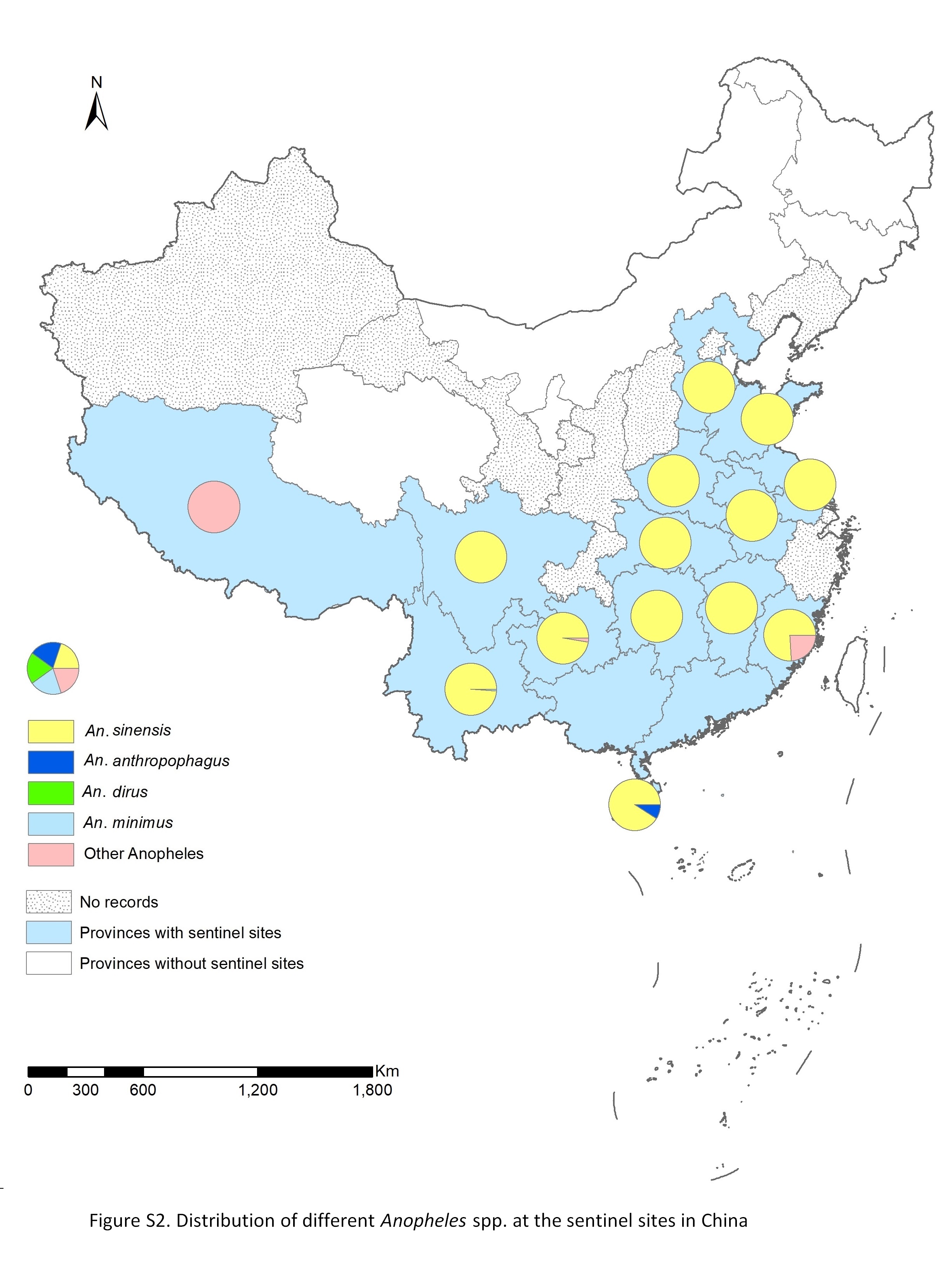

Supplement: Supplemental Material [file TEMI_A_2026740_SM0818.zip › Suppl files/Supplemental file 3.jpg]
